# Backpacks Effect on Foot Posture in Schoolchildren with a Neutral Foot Posture: A Three-Year Prospective Study

**DOI:** 10.3390/ijerph17197313

**Published:** 2020-10-07

**Authors:** Pilar Alfageme-García, Julián Fernando Calderón-García, Alfonso Martínez-Nova, Sonia Hidalgo-Ruiz, Mariana Martínez-Álvarez, Sergio Rico-Martín

**Affiliations:** 1 Nursing Department, University of Extremadura, 10003 Cáceres, Spain; palfagemeg@unex.es (P.A.-G.); podoalf@unex.es (A.M.-N.); kirosony@unex.es (S.H.-R.); marianama@unex.es (M.M.-Á.); sergiorico@unex.es (S.R.-M.); 2 Nursing Departament, University Center of Plasencia, University of Extremadura, 10600 Plasencia, Spain; 3 Nursing and Occupational Therapy College, University of Extremadura, 10003 Cáceres, Spain; 4 Faculty of Medicine, University of Extremadura, 06006 Badajoz, Spain

**Keywords:** flat foot, foot index posture, backpack, schoolchildren, neutral foot, supinated foot, pronated foot

## Abstract

Background: There is a paucity of data on the relationship between backpack use and foot posture in children. The aim of this study was to assess the effects of a backpack on foot posture in children with neutral foot posture during three years of follow-up. Methods: A prospective longitudinal observational study was conducted in a sample of 627 children with neutral foot. For each participant included in the study, age, sex, weight, height, body mass index, type of schoolbag (backpack or non-backpack), foot shape, metatarsal formula and type of shoes were recorded. Foot posture was described by the mean of the foot posture index (FPI) and reassessed after three years in a follow-up study. Results: The average age of the children was 8.32 ± 1.32 years. A total of 458 used a backpack when going to school. Over the three-year follow-up period, 50 children who had neutral foot developed supinated foot (*n* = 18) or pronated foot (*n* = 32). Univariate and multivariate analysis showed that the children using a backpack were at a higher risk of developing pronated foot (adjusted Odds Ratio (aOR) = 2.05, 95% IC: 1.08–3.89, *p* = 0.028). Backpack use was not associated with the change from neutral foot to supinated foot. Conclusions: We found a positive association between using a backpack and the risk of developing pronated but not supinated foot. Clinical trials should be conducted to analyze the effect of backpack use on the foot among schoolchildren.

## 1. Introduction

The foot is a functional unit of the human body that plays a key role in movement and balance [1,2]. The morphological and functional development of a child’s feet can be influenced by internal (sex, age and genetics) and external factors (shoes, body weight and physical activity) [3,4,5,6].

Flatfoot is a complex foot deformity commonly observed in children, and it frequently concerns parents [7]. The definition of flatfoot is not standardized; however, flatfoot is characterized by a pronated foot demonstrating collapse of the medial longitudinal arch, foot abduction at the talonavicular joint, and hindfoot valgus (subtalar joint eversion) [8,9]. Many children have physiologic flatfoot, which is almost uniformly asymptomatic and flexible [10]. Some cases of flatfoot can negatively impact quality of life [11] lead to comorbidities [12]. Moreover, flatfoot can be associated with lower extremity injuries [13] and other foot problems, including foot pain [14,15], hallux abducto valgus [16,17], hammertoes [18], and osteoarthritis [19]. The treatment of asymptomatic pediatric flatfoot has been discussed to a great extent [20]. However, there is no evidence that orthoses either correct deformities or prevent the development of symptoms [10,21]. In patients with asymptomatic flexible flatfoot, management often requires only monitoring foot development and parental education [10,21].

It is generally believed that to some extent, the incidence of flexible flatfoot [22] as a physiological condition decreases with age [23]. It is a normal evolutionary phenomenon that children have flatfoot when they start walking, as intrinsic ligamentous laxity and the lack of neuromuscular control result in a flattening of the foot under load [24]. In children aged between 1 and 5 years, there is an increased thickness of fat under the medial longitudinal plantar arch, which disappears after the age of approximately 5 years, when the foot structure approaches its mature form [3]. Different procedures can be used to diagnose flatfoot [25,26,27,28,29,30]. Static foot assessments are commonly performed in clinical practice to classify the foot type, identify possible etiological factors related to injury and prescribe therapeutic interventions [31]. The foot posture index (FPI) [25] is an observational assessment tool that takes into account the three-dimensional nature of the posture of the foot and has demonstrated good reliability in children [6,32,33,34]. The FPI is a valid tool for diagnosing pronated foot or flatfoot [25,35].

Every day, most children spend a great deal of time carrying a backpack containing school materials, and the backpack is sometimes too heavy. The angle of forward lean of the trunk is larger during load carriage on the back compared with during normal gait [36], and load carriage on the back is related to back pain [37]. It is well known that the heavier the load is, the higher the pressure and force under the different regions of the foot [38], and load carriage can alter an individual’s gait pattern [39,40,41]. Postural changes have been observed under both static and dynamic walking conditions, with external loads exceeding 20% of the child’s body mass [42].

Backpack effects on foot structure and functionality have been investigated only in soldiers, where backpack use was associated with an increase in the trunk flexion angle, an increase in hip and ankle range of motion, an increase in the vertical and horizontal ground reaction forces, an increase in cadence and a decrease in stride length [43]. Backpack carriage increases peak plantar pressures, which are already elevated in children, during upright stance and modifies the physiologic pressure patterns, especially in the forefoot area and during static conditions [38,44].

However, no previous studies have investigated the relationship between backpack use and foot posture in children. The aim of this study was to assess the effect of backpacks use on foot posture in children with a neutral foot posture over a follow-up period of three years.

## 2. Materials and Methods

### 2.1. Study Design, Setting and Participants

**Figure 1:**
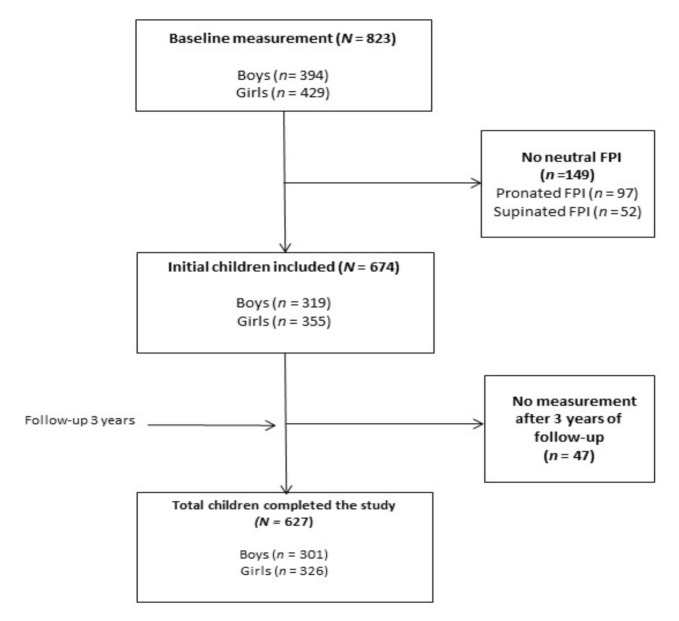
Participant selection process.

A prospective longitudinal observational study was conducted in all (a total of 15) primary schools in Plasencia (Spain). We aimed to have sufficient statistical power to detect medium-to-small effect sizes (anticipated Cohen’s d = 0.15) with β = 0.95 and α = 0.05, which required a minimum sample size of 580 participants [45]. Finally, the study population consisted of 627 children, including 301 boys (48.0%) and 326 girls (52.0%), with an age range between five and eleven years (mean age, all: 8.3 ± 1.6 years; boys: 8.2 ± 1.6; girls: 8.3 ± 1.6). Recruitment began in March 2014 and finished in June 2014. Figure 1 illustrates the participant selection process. A total 823 schoolchildren were evaluated (of these 149 were excluded because they did not have a neutral foot). We included a total of 674 schoolchildren in this study; however, the measures were not repeated for 47 (7%) schoolchildren, so they were excluded.

The criteria for inclusion were as follows: (1) an FPI score indicating neutral foot; (2) asymptomatic feet; (3) symmetrical feet, with no evident joint deformities and (4) an age of five to eleven years. The exclusion criteria were as follows: (1) an FPI score indicating pronation or supination of the foot, (2) foot pain, (3) injury to the lower limbs during the previous six months, (4) congenital abnormalities, (5) neurological diseases, (6) a history or foot surgery and (7) the use of foot orthoses. A total of 149 of the initial participants did not have FPI scores indicating neutral foot, and 47 were lost to follow-up and subsequently excluded from the study (due to lower-limb injury occurring within the three years).

This study was reported following the STrengthening the Reporting of OBservational studies in Epidemiology (STROBE) guidelines for reporting observational studies [46] and was designed in accordance with the Declaration of Helsinki. The study protocol was approved by the ethics committee of the University of Extremadura (ID: 59/2012). The parents of the participants were informed about the details of the study and asked to complete a questionnaire and to sign a consent form allowing their children to participate in the study.

### 2.2. Study Variables

For each participant included in the study, age, sex, type of schoolbag (backpack or non-backpack) and type of shoes were recorded. Height was measured using a Harpenden stadiometer, and weight was measured using a biomedical precision balance. Body mass index (BMI) was calculated as the weight in kilograms divided by the square of the height in meters (kg/m^2^). The subjects were classified into the following four categories according to the BMI range corresponding to their age using the classification system proposed by Orbegozo [47]: underweight, normal, overweight and obese. Children who were not physically active outside of school were considered sedentary. The podiatric medical examination, performed by an experienced podologist, included the assessment of the metatarsal formula and foot shape. The metatarsal formula [48] was classified as index plus (the first metatarsal was longer than the second, and the remaining metatarsals progressively decreased in size), index minus (the first metatarsal was the same length as the second, and the remaining metatarsals progressively decreased in size) or index plus minus (the first metatarsal was shorter than the second, and the remaining metatarsals progressively decreased in size). Foot shape [48] was classified as Egyptian (the great toe was the longest), Greek (the second toe was the longest) or square (the great toe and second toe were the same length).

### 2.3. FPI Measurement

The FPI was evaluated using the standard method [25]. The same examiner made all measurements. The FPI was assessed with the children in a relaxed stance on a bench that was 50 cm tall. The same protocol that has been reported in other studies [6,49,50,51] was used. The FPI reflects the multisegmental nature of the posture of the foot in all three planes and does not require the use of specialized equipment. The six criteria of this index were evaluated: (1) talar head palpation, (2) supra- and infra-malleolar curvature, (3) calcaneal frontal plane position, (4) prominence in the region of the talonavicular joint, (5) congruence of the medial longitudinal arch and (6) abduction/adduction of the forefoot on the rearfoot. Each item of the FPI was scored between −2 and +2. The total FPI score may range from −12 to +12, and foot posture can be classified as highly supinated (−12 to −5), supinated (−4 to −1), neutral (0 to 5), pronated (6 to 9), or highly pronated (10 and 12) [33].

To preserve independence of the data [52] and given that there is a strong correlation between the FPI scores of the left and right feet in healthy individuals [32], the data for only one foot (the right, chosen at random) were included in the statistical analyses, although the data for both feet were measured.

### 2.4. Prospective Assessment

Anthropometric and foot posture assessments were performed at baseline and, with a prospective intent, repeated three years later by the same investigator (PAG) with the same protocol.

### 2.5. Statistical Methods

Statistical analysis was performed using IBM SPSS, version 24 (IBM Corporation, Armonk, NY, USA). The continuous variables are expressed as the mean ± standard deviation, and the categorical variables are expressed as percentages. The Kolmogorov–Smirnov test was used to determine whether the variables followed a normal distribution. Scores of six criteria of FPI and total score FPI were not normal distribution. The continuous variables were compared using Student’s t-test. Individual characteristics were analyzed using Student’s t-test (independent samples, backpack vs. non-backpack; paired samples, first and last recorded scores) or non-parametric tests (independent-samples Mann–Whitney U test; paired-samples Wilcoxon test), as appropriate. The categorical variables were compared using the chi-square test or Fisher’s exact test.

Univariate and multivariate logistic regression analyses were performed to assess the association between the independent variables and the change from neutral foot to pronated or supinated foot within the 3 years of follow-up (dependent variable). Odds ratios and the corresponding 95% confidence intervals were calculated. All variables with a significance level of <0.05 in univariate analysis were considered for inclusion in the multivariate analysis. The variables included as predictors/confounders were age, sex, backpack use, the presence/absence of overweight-obesity, the presence/absence of a sedentary status, metatarsal formula, foot shape and shoe type. The level of statistical significance was set to be *p* < 0.05.

## 3. Results

A total of 627 children were recruited (301 boys and 326 girls). Of these children, 458 (73%) used a backpack when going to school. The average age was 8.32 ± 1.61 years, with a range from 5 to 11 years, and the average BMI was 18.76 ± 3.74 kg/m^2^. The mean FPI score at baseline was 2.42 ± 1.55, with the total score ranging from 0 to 5. The main characteristics of the backpack and non-backpack participants are shown in Table 1. There were significant differences between groups in age (*p* < 0.001), the presence of overweight-obesity (*p* = 0.016) and the use of sport- (*p* = 0.026) and moccasin-type (*p* = 0.001) shoes.

Table 2 shows the longitudinal study results for the total and FPI scores for each of the six criteria in all children and stratified by the backpack and non-backpack groups. In all participants, after three years, the total FPI score (*p* = 0.042) and the scores for two (of six) FPI criteria, talar head palpation (*p* = 0.01) and abduction/adduction of the forefoot on the rearfoot significantly decreased (*p* = 0.03). In the backpack group, the FPI scores significantly decreased for two FPI criteria: talar head palpation (*p* = 0.003) and abduction/adduction of the forefoot on the rearfoot (*p* = 0.011). However, there was no significant difference in any FPI criterion in the non-backpack group from baseline to the 3-year follow-up.

Over the three-year follow-up period, a total of 50 children who had neutral foot developed supinated foot (*n* = 18) or pronated foot (*n* = 32). The predictors for the change from neutral foot to pronated foot are shown in Table 3. According to the univariate analysis, the children using backpacks were at a higher risk of developing pronated foot (odds ratio (OR): 5.90; 95% CI: 1.39–24.99). Moreover, the sedentary children (OR: 3.00; 95% CI: 1.44–6.27) and girls using Mary Jane shoes (OR: 3.78; 95% CI: 1.21–11.76) had a significantly higher risk of developing pronated foot. The multivariate analysis yielded similar results, where the children using a backpack (aOR: 6.44; 95% CI: 1.49–27.82), sedentary children (aOR: 2.86; 95% CI: 1.35–6.06) and girls using Mary Jane shoes (aOR: 4.19; 95% CI: 1.27–13.78) had a significantly high risk of developing pronated foot. Table 4 shows the predictors for the change from neutral foot to supinated foot. According to the univariate analysis, the children with a metatarsal formula index of plus minus were at a lower risk of developing supinated foot (OR: 0.31; 95% CI: 0.11–0.89). In contrast, the children with a metatarsal formula index of minus were at a higher risk (OR: 3.43; 95% CI: 1.32–8.90). However, in the multivariate analysis, no variables were associated with the change from neutral to supinated foot. Finally, the use of backpack was not shown to be a risk factor for supinated foot.

## 4. Discussion

In this cohort of Spanish children, we examined the association between the use of a backpack and changes in foot posture in children aged 5 to 11 years with neutral foot posture over a follow-up period of three years. Our results indicated that backpack use may be associated with the development of pronated but not supinated foot in children with neutral foot.

Supination and pronation are complex joint movements that occur in all three planes [53]. Supination involves plantar flexion, adduction, inversion and inversion movements of the ankle joint, subtalar joint and Chopart/Lisfranc joint. On the other hand, pronation involves dorsiflexion in the sagittal plane, eversion in the frontal plane and abduction in the transverse plane, which are achieved by the articulation of the foot in many degrees of freedom.

The use of backpacks in schoolchildren is very common. In our study, more than 70% of schoolchildren were found to carry backpacks, and they probably carry loads higher than the recommended limit, so load carriage in children has public health implications [54]. Postural changes have been observed in both static and dynamic gait, with external loads exceeding 20% of the child’s body mass [42]. It is well known that the heavier the load is, the higher the pressure and force under the different regions of the foot [38], and it is known that load carriage can alter an individual’s gait pattern [39,40,41]. Some authors even claim significant peak and mean pressures in the forefoot during standing and static conditions [54,55]. These findings have been similarly common in obese and nonobese schoolchildren [56].

On the other hand, Al-Khabbaz observed that the muscular activity and position of the trunk depend on the weight of the backpack in university students [57]. A study of the muscular activity of the rectus, erector, biceps femoral, and vastus medial muscles showed that the muscular activity of the rectus abdominal muscle increases significantly when loads greater than 10% of body weight are applied.

Logistic regression showed that age, sex, BMI, metatarsal formula and foot shape had no influence on the change from neutral to pronated foot in the schoolchildren studied. However, backpack use, sedentariness and Mary Janes shoe use (in girls) did influence this change, according to both the univariate and multivariate analyses. This longitudinal study assessed the changes that can occur in schoolchildren with neutral foot and due to a paucity of data in the field of the current study, there are no studies to compare our findings with other research. Our results showed that there is a relationship between backpack use and the development of flatfoot or pronated foot but not supinated foot over a follow-up period of 3 years in children who previously had neutral foot. This relationship can be explained by the fact that backpack carriage during walking is associated with an increased trunk flexion angle, increased hip and ankle ranges of motion, increased vertical and horizontal ground reaction forces, increased cadence and a reduced stride length [43].

In contrast to our results, the results of cross-sectional studies have shown a relationship between BMI and pronated foot; however, the analysis performed was based on a single plane and used a different measurement element, the footprint [4,58,59,60,61]. We assessed the FPI, which reflects the foot posture in all three spatial planes and considers the three functional units of the foot (hindfoot, midfoot and forefoot). Finally, the development of flatfoot can lead to sedentary behavior in these children due to difficulty performing physical activity. However, there are no studies showing that sedentary behavior can influence the development of flatfoot, as our results showed.

This longitudinal study has several limitations. First, the design of the study was observational and therefore can only indicate associations and not causality. Moreover, this study reports measures only at baseline and after three years. Most likely, annual assessments may have provided more information and precision regarding when the pediatric foot posture changes developmentally. Moreover, all of the children were recruited in our region and surrounding areas; thus, the findings may not be applicable to other populations. Finally, because our study was not designed to analyze the effect of backpack use on the foot, we did not record the usual weight of the backpacks, as it is known that a higher percentage of load causes larger postural changes [42] and higher pressures and forces under the different regions of the foot [38]. In addition, we did not consider the duration of use of the backpacks and their positions on the back.

## 5. Conclusions

In conclusion, we found a positive association between using backpacks and the risk of developing pronated but not supinated foot over a follow-up of three years in children aged 5 to 11 years with neutral foot posture. Clinical trials should be conducted to analyze the effect of backpack use on the foot among schoolchildren.

## Figures and Tables

**Table 1:** Biological, anthropometric, sociocultural and foot characteristics of the children stratified by backpack use.

Children Characteristics	All (*n* = 627)	Backpack (*n* = 458)	Non-Backpack (*n* = 169)	*p*-Value
**Age (months)**	8.32 ± 1.61	8.68 ± 1.60	7.33 ± 1.18	<0.001
**Age categorized, years (%)**				
<7 years	159 (25.4%)	83 (18.1%)	76 (45.0%)	<0.001
7–9 years	318 (50.7%)	232 (50.7%)	86 (50.9%)	0.959
>9 years	150 (23.9%)	143 (31.2%)	7 (4.1%)	<0.001
**Gender male (%)**	301 (48.0%)	220 (48.0%)	81 (47.9%)	0.981
**BMI (kg/m^2^)**	18.76 ± 3.74	18.74 ± 4.54	18.80 ± 4.54	0.875
**Overweight-obesity (%)**	42 (4.7%)	24 (5.2%)	18 (10.7%)	0.016
**Sedentary (%)**	126 (20.1%)	95 (20.7%)	31 (18.3%)	0.506
**Metatarsal formula (%)**				
Index Plus	163 (26.0%)	120 (26.2%)	43 (25.4%)	0.848
Index Plus Minus	343 (54.7%)	251 (54.8%)	92 (54.4%)	0.935
Index Minus	121 (19.3%)	87 (19.0%)	34 (20.1%)	0.752
**Foot shape (%)**				
Greek foot	150 (23.9%)	109 (23.9%)	41 (24.3%)	0.904
Square foot	372 (59.3%)	275 (60.0%)	97 (57.4%)	0.549
Egyptian foot	105 (16.7%)	74 (16.2%)	31 (18.3%)	0.515
**Shoe type**				
Sport	411 (65.6%)	312 (68.1%)	99 (58.6%)	0.026
Moccasin	64 (10.2%)	36 (7.9%)	28 (16.6%)	0.001
Boot	16 (2.6%)	9 (2.0%)	7 (4.1%)	0.125
Ballet flats	110 (17.5%)	84 (18.3%)	26 (15.4%)	0.388
Mary Janes	26 (4.1%)	17 (3.7%)	9 (5.3%)	0.369

Data expressed as mean ± standard deviation and frequencies (percentages). Abbreviations: BMI: Body mass index.

**Table 2:** Differences between the total FPI scores and those for the six index criteria according to backpack use.

FPI	All			Backpack			Non-Backpack		
	Initial	Repeat	*p*-Value	Initial	Repeat	*p*-Value	Initial	Repeat	*p*-Value
Talar head palpation	0.69 ± 0.50	0.61 ± 0.53	0.01	0.70 ± 0.50	0.62 ± 0.54	0.003	0.65 ± 0.50	0.57 ± 0.50	0.090
Curves at malleolus	0.30 ± 0.49	0.28 ± 0.49	0.526	0.29 ± 0.48	0.29 ± 0.49	0.999	0.30 ± 0.52	0.24 ± 0.48	0.250
Inversion/Eversion calcaneus	0.31 ± 0.49	0.29 ± 0.49	0.682	0.29 ± 0.49	0.29 ± 0.49	0.999	0.35 ± 0.50	0.31 ± 0.49	0.440
TNJ prominence	0.30 ± 0.51	0.33 ± 0.48	0.166	0.32 ± 0.51	0.33 ± 0.48	0.618	0.25 ± 0.51	0.33 ± 0.49	0.080
5 Congruence of medial arch	0.37 ± 0.52	0.36 ± 0.52	0.843	0.38 ± 0.51	0.37 ± 0.50	0.874	0.34 ± 0.55	0.33 ± 0.56	0.906
Ab/adduction forefoot-rearfoot	0.48 ± 0.54	0.43 ± 0.50	0.034	0.51 ± 0.54	0.43 ± 0.50	0.011	0.41 ± 0.52	0.42 ± 0.50	0.899
Total SCORE FPI	2.42 ± 1.55	2.31 ± 1.64	0.042	2.47 ± 1.54	2.36 ± 1.66	0.070	2.30 ± 1.59	2.20 ± 1.60	0.354

Data expressed as mean ± standard deviation. Abbreviations: Ab: abduction; FPI: Foot posture index; TNJ: Talonavicular joint.

**Table 3:** Predictors of the changes from neutral foot to pronated foot.

Children Characteristics			Univariate Analysis		Multivariate Analysis	
	FPI Pronated *N* = 32	FPI Neutral *N* = 577	OR (95% CI)	*p*-Value	aOR (95% CI)	*p*-Value
**Backpack**	30 (93.8%)	414 (71.8%)	5.90 (1.39–24.99)	0.016	6.44 (1.49–27.82)	0.013
**Age (months)**	-	-	1.00 (0.98–1.02)	0.709	-	-
**Age categorized**						
Age <7 years	7 (21.9%)	146 (25.3%)	0.82 (0.35–1.95)	0.663	-	-
Age 7–9 years	16 (50.0%)	294 (51.0%)	0.96 (0.47–1.96)	0.916	-	-
Age >9 years	9 (28.1%)	137 (23.7%)	1.25 (0.56–2.78)	0.572	-	-
**Gender male (%)**	16 (50.0%)	299 (51.8%)	0.93 (0.45–1.89)	0.841	-	-
**BMI (kg/m^2^)**	-	-	1.01 (0.93–1.11)	0.701	-	-
**Overweight-obesity (%)**	1 (3.1%)	39 (6.8%)	0.44 (0.05–3.34)	0.714	-	-
**Sedentary (%)**	13 (40.6%)	107 (18.5%)	3.00 (1.44–6.27)	0.003	2.86 (1.35–6.06)	0.006
**Metatarsal formula (%)**						
Index Plus	7 (21.9%)	151 (26.2%)	0.79 (0.33–1.86)	0.590	-	-
Index Plus Minus	21 (65.6%)	317 (54.9%)	1.56 (0.74–3.30)	0.236	-	-
Index Minus	4 (12.5%)	109 (18.9%)	0.61 (0.21–1.78)	0.486	-	-
**Foot shape (%)**						
Greek foot	8 (25.0%)	135 (23.4%)	1.01 (0.47–2.48)	0.835	-	-
Square foot	20 (62.55)	344 (59.6%)	1.12 (0.54–2.35)	0.746	-	-
Egyptian foot	4 (12.5%)	98 (17.0%)	0.69 (0.24–2.03)	0.632	-	-
**Shoe type**						
Sport	17 (53.1%)	379 (65.7%)	0.59 (0.29–1.21)	0.147	-	-
Moccasin	3 (9.4%)	60 (10.4%)	0.89 (0.26–3.01)	0.999	-	-
Boot	0 (0.0%)	16 (2.8%)	-	0.340	-	-
Ballet flats	8 (25.0%)	101 (17.5%)	1.57 (0.68–3.59)	0.282	-	-
Mary Janes	4 (12.5%)	21 (3.6%)	3.78 (1.21–11.76)	0.022	4.19 (1.27–13.78)	0.018

Data expressed as frequencies (percentages) and OR (95% CI). Abbreviations: aOR: adjusted Odds Ratio; BMI: Body mass index; CI: Confidence interval; FPI: Foot posture index; OR: Odds Ratio.

**Table 4:** Predictors of the changes from neutral foot to supinated foot.

Children Characteristics			Univariate Analysis		Multivariate Analysis	
	FPI Supinated *n* = 18	FPI Neutral *n* = 577	OR (95% CI)	*p*-Value	aOR (95% CI)	*p*-Value
**Backpack**	14 (77.8%)	414 (71.8%)	1.30 (0.42–4.00)	0.575	-	-
**Age (months)**			0.99 (0.96–1.01)	0.562	-	-
**Age categorized**						
Age <7 years	6 (33.3%)	146 (25.3%)	1.47 (0.54–4.00)	0.442	-	-
Age 7–9 years	8 (44.4%)	294 (51.0%)	0.77 (0.33–1.97)	0.586	-	-
Age >9 years	4 (22.2%)	137 (23.7%)	0.91 (0.29–2.83)	0.881	-	-
**Gender male (%)**	7 (38.9%)	278 (48.2%)	0.68 (0.26–1.79)	0.437	-	-
**BMI (kg/m^2^)**			0.99 (0.87–1.12)	0.881	-	-
**Overweight-obesity (%)**	2 (11.1%)	39 (6.8%)	0.58 (0.12–2.61)	0.356	-	-
**Sedentary (%)**	6 (33.3%)	107 (18.5%)	2.19 (0.80–5.98)	0.115	-	-
**Metatarsal formula (%)**						
Index Plus	5 (27.8%)	151 (26.2%)	1.08 (0.38–3.09)	0.793	-	-
Index Plus Minus	5 (27.8%)	317 (54.9%)	0.31 (0.11–0.89)	0.029	0.47 (0.13–1.67)	0.247
Index Minus	8 (44.4%)	109 (18.9%)	3.43 (1.32–8.90)	0.007	2.21 (0.70–6.96)	0.173
**Foot shape (%)**						
Greek foot	7 (38.9%)	135 (23.4%)	2.08 (0.79–5.48)	0.129	-	-
Square foot	8 (44.4%)	344 (59.6%)	0.54 (0.21–1.39)	0.197	-	-
Egyptian foot	3 (16.7%)	98 (17.0%)	0.97 (0.27–3.44)	0.999	-	-
**Shoe type**						
Sport	15 (83.5%)	379 (65.7%)	2.61 (0.74–9.13)	0.119	-	-
Moccasin	1 (5.6%)	60 (10.4%)	0.50 (0.06–3.87)	0.505	-	-
Boot	0 (0.0%)	16 (2.8%)	-	0.474	-	-
Ballet flats	1 (5.6%)	101 (17.5%)	0.27 (0.03–2.10)	0.336	-	-
Mary Janes	1 (5.6%)	21 (3.6%)	1.55 (0.19-)	0.671	-	-

Data expressed as frequencies (percentages) and OR (95% CI). Abbreviations: aOR: adjusted Odds Ratio; BMI: Body mass index; CI: Confidence interval; FPI: Foot posture index; OR: Odds Ratio.
